# Probiotic Bacteria Influence the Composition and Function of the Intestinal Microbiota

**DOI:** 10.1155/2008/175285

**Published:** 2008-12-03

**Authors:** Paul W. O'Toole, Jakki C. Cooney

**Affiliations:** ^1^Department of Microbiology and Alimentary Pharmabiotic Centre, University College Cork, Cork, Ireland; ^2^Department of Life Sciences and Materials and Surface Sciences Institute, University of Limerick, Limerick, Ireland

## Abstract

Probiotics have a range of proposed health benefits for the consumer, which may include modulating the levels of beneficial elements in the microbiota. Recent investigations using molecular approaches have revealed a human intestinal microbiota comprising over 1000 phylotypes. Mechanisms whereby probiotics impact on the intestinal microbiota include competition for substrates, direct antagonism by inhibitory substances, competitive exclusion, and potentially host-mediated effects such as improved barrier function and altered immune response. We now have the microbial inventories and genetic blueprints to begin tackling intestinal microbial ecology at an unprecedented level of detail, aided by the understanding that dietary components may be utilized differentially by individual phylotypes. Controlled intervention studies in humans, utilizing latest molecular technologies, are required to consolidate evidence for bacterial species that impact on the microbiota. Mechanistic insights should be provided by metabolomics and other analytical techniques for small molecules. Rigorous characterization of interactions between the diet, microbiota, and probiotic bacteria will provide new opportunities for modulating the microbiota towards improving human health.

## 1. INTRODUCTORY REMARKS

The history of microbiological research has been dominated by investigations of the agents of human infectious disease. Motivated by the desire to culture, characterize, and understand the pathogenicity mechanisms of these organisms, several centuries of microbiological research culminated in a broad range of antimicrobial therapies, vaccines, and immunizations. In more recent years, similar analytical methodologies have been applied to facilitate exploitation of bacteria for industrial applications. Two related branches of microbiology—environmental microbiology, and the study of intestinal commensals (a branch of the first in purest terms)—lagged behind until relatively recently. From the mid 1990s, a range of techniques allowed environmental microbiologists to indentify soil microorganisms *in situ*, without resorting to culture, based upon ribosomal small subunit RNA gene probes. A natural extension of this approach was to sequence large numbers of cloned ribosomal RNA gene amplicons, yielding catalogs of all the organisms (the microbiota) present in complex samples. Latterly the field of metagenomics has provided technical approaches to sequence large fractions of the entire microbial DNA present in an ecological system. Coupled with the application of molecular tools for studying commensal bacteria, many of which were originally developed for studying pathogens, there is now an exciting nexus between technologies and research foci whereby commensal bacteria may be studied in the context of intestinal ecosystems. This review will summarize what is known about the effect of introducing probiotic bacteria on the composition and activities of the microbiota, with an emphasis on recent studies using culture-independent methods. The likely mechanisms whereby commensals exert their influence are discussed, and directions for future research are outlined.

## 2. THE CONCEPTS OF PROBIOTICS AND PREBIOTICS

The notion that certain intestinal microorganisms might benefit the host derives historically from suggestions by Metchnikoff and others that putrefying bacteria that contribute to toxification and aging could be deliberately replaced by fermentative organisms [1, 2]. In this context, some of the fermentative bacteria, Metchnikoff was referring to, are what we now consider as probiotic. Probiotic bacteria are live microorganisms which when administered in adequate amount confer a health benefit on the host [3, 4]. Many microorganisms that are considered probiotic have been traditionally used to preserve food products by fermentation, and are present in the food in varying numbers, along with their fermentation end products and other metabolites. Thus another operational definition of the term probiotic requires the organism in question to be “consumed in adequate amounts” to confer a benefit [5]. The host benefits that have been attributed to consumption of probiotic microorganisms are diverse (reviewed in [3, 6–8]; some major examples are listed in Table 1), and have been substantiated to different degrees. Probiotic bacteria are now included in a wide range of consumer formulations including yoghurts, drinks, capsules, and dietary supplements, and they represent a significant element in the modern functional foods market. Organisms used as probiotic agents are frequently members of the genera *Lactobacillus* or *Bifidobacterium*, but *Escherichia coli*, *Bacillus subtilis*, *Saccharomyces boulardii,* and *Enterococcus faecium* are also employed, among others [9]. Thus, an organism employed as a probiotic agent may not necessarily be part of what is considered the “normal microbiota.” Tannock distinguishes between allochthonous and autochthonous species [10, 11]. Autochthonous means bacteria both present and replicating *in situ* in the human GI tract, as distinct from transiently passing through (allochthonous). Bacteria administered as probiotic agents are not necessarily autochthonous to the consuming animal, and indeed some *Lactobacillus* species may only be autochthonous for certain human individual subjects, and possibly not the majority of subjects (see below). Rate of growth of allochthonous lactobacilli may be a critically limiting step preventing their establishment [12]. With regard to developing probiotic strains for exploitation, it may prove easier to identify beneficial traits in species that are autochthonous to the human consumer, as consumer acceptance is likely to be easier if the probiotic ingredient in a functional food (a food product with benefit to the consumer over and above inherent nutrition) was first cultured from humans.

Related to the consumption of probiotic agents is the notion of dietary adjustment to stimulate bacterial growth. A prebiotic compound is defined as “a nondigestible food ingredient that beneficially affects the host by selectively stimulating the growth and/or activity of one or a limited number of bacteria in the colon and thus improves host health” [30, 31]. It follows from this definition that the bacteria capable of metabolizing prebiotics should be restricted to a small number of beneficial species or strains (reviewed in [32]). In practice, prebiotic compounds must also be refractory to host digestive processes, and the combined catabolic activities of bacteria higher up in the gastrointestinal tract, so that prebiotic compounds are often oligosaccharides towards which probiotic bacteria produce specific hydrolases [33]. Prebiotics are commonly found in, or extracted from, plant material including fruits, cereal, and vegetables, but are also present in human milk and colostrum [6]. The best characterized prebiotics include inulin, fructooligosaccharide, galactooligosaccharide, xylooligosaccharide, isomaltooligosaccharide, and lactulose (reviewed in [32]). Unravelling the health benefits of prebiotics is a challenging task, because these compounds have parallel direct effects on the host, and potentially on multiple members of the microbiota. For example, *β*-glucans are unbranched polysaccharides with (1-4) and (1-3)-linked *β*-D glucopyranosyl units, that are recognized as important dietary ingredients (reviewed in [34]). *β*-glucans are components of plant cell walls, and are abundant in the endosperm of cereals such as barley and oatmeal. Consumption of *β*-glucans has attendant health benefits that are recognized by health and regulatory bodies in several jurisdictions including the US [35]. These benefits include lowering of blood cholesterol and lipoprotein [36], lowering of postprandial glucose and insulin responses [37], and enhancement of antitumor monoclonal antibodies [38]. Supplementation of mammalian diet with *β*-glucan, or modification by prehydrolysis of *in vitro* bacterial growth medium, leads to increased numbers and proportions of lactobacilli or bifidobacteria [39–41]. Furthermore, *β*-gluco-oligomers promoted the growth of *L. rhamnosus* GG [40]. Barley supplementation of rat diet [39] led to an increase in *Lactobacillus* numbers, a decrease in *Bacteroides* and coliforms, and an increase in the production of butyrate. Butyrate is an important energy source, signalling metabolite, proliferation stimulus for normal colonic epithelial cells, and anti-proliferative signal for neoplastic colonocytes [42, 43], suggesting a potential direct benefit from dietary ingredients or prebiotics that promote growth of clostridia.

## 3. THE NORMAL MICROBIOTA OF HUMANS AND ANIMALS

Until recently, the composition of the microbiota was examined by relatively insensitive techniques. Culturing the bacteria was unrepresentative, because a large proportion of the bacteria do not grow on standard laboratory media [44]. Analysis by temperature gradient gel electrophoresis provided one of the earliest insights into the uncharted complexity of the microbiota [45]. Using denaturing gradient gel electrophoresis (DGGE) of 16S rRNA gene amplicons, the same group later showed that the colonic mucosal microbiota and faecal microbiota were different, and the colonic mucosal microbiota was likely dependent on host factors [46]. Meanwhile fluorescent hybridization of probes for 16S rRNA genes was being applied to determine species identities, numbers, and proportions of intestinal bacteria [47, 48], exemplified by the studies of Dore, Blaut and colleagues [49, 50]. These analyses highlighted extensive inter-individual variation at phylotypes level (among northern Europeans) [49], and some correlations of microbiota with age, gender, and geographic origin but which varied between countries [50].

Our current understanding of the intestinal microbiota (reviewed in [51, 52]) has been significantly shaped by culture-independent methods, in particular the sequencing of 16S rRNA gene amplicons, either from clone libraries or direct pyrosequencing of the PCR product. A consensus appears to be emerging in the literature of somewhere between 800 and 1000 bacterial phylotypes being present in the healthy human intestine [52]; the evidence for which will be selectively presented here. A relatively small-scale investigation by Benno and colleagues in 2003 revealed an unexpectedly high number of novel phylotypes in 240 partially sequenced 16S rRNA gene amplicons clones derived from six elderly individuals [53]. In a pioneering study, Relman and colleagues applied the 16S rRNA molecular inventory-based approach, at a much larger scale than previously published, on samples from both colonic sites and faeces [54]; strikingly, the majority of the sequences derived corresponded to uncultivated species and novel microorganisms. The human stomach, previously considered sterile except for infections with *Helicobacter pylori*, was revealed by 16S rRNA gene library sequencing to be well populated by bacteria, based on detection of 128 bacterial phylotypes from 23 gastric endoscopy samples [55]. Gill and colleagues [56] sequenced not just 16S rRNA genes, but also randomly cloned bacterial DNA—so-called *metagenomics*, a concept developed for environmental community analysis [57]. Gill et al. showed by this metagenomic approach that the bacteria in the gut significantly expand the metabolic capabilities of the human gut [56]. By generating two to three 454 pyrosequencing runs per mouse cecum, Gordon and colleagues showed the existence of an obesity-associated gut microbiome with increased capacity for energy harvest [58]. Significantly, this balance of the microbiota was borne out when investigating obese humans [59], showing a seminal link between human obesity and changes in the microbiota. Furthermore, the complexity of the microbiota in humans and 59 other mammalian species was shown to be linked to phylogeny (of the mammal) and the composition of the diet [60]. Analysis of the metabolic capability likely conferred by the microbial metagenome recently revealed 237 gene families commonly enriched in adult-type and 136 families in infant-type microbiomes [61]. Thus, any consideration of the effect of probiotics on the intestinal metagenome should ultimately include analysis of the downstream effects upon the host of impacting on this metabolic capability. A more fundamental consideration is that the genera whose members are among the most commonly employed probiotics—*Bifidobacterium* and *Lactobacillus*—are not present in the human gastrointestine at the high levels traditionally expected based on culture-based approaches, being represented by 20 phylotypes (ca. 2%) and 36 phylotypes (ca. 3.6%), respectively [52]. Thus if probiotic bacteria impart health benefits to the host under “natural conditions,” that is, in individuals who have normal nonmanipulated numbers of probiotic bacteria, they accomplish this despite being at much lower numbers than are achieved by consumption of probiotic products.

## 4. PROBIOTICS AND THEIR EFFECT UPON THE MICROBIOTA

There have been relatively few studies which have rigorously characterized the effect upon the whole microbiota of administering probiotic cultures, and until recently, all such studies applied targeted analysis of specific groups of bacteria. In one of the earliest investigations, Tannock and colleagues observed transient and modest fluctuations in lactobacillus and bifidobacterium numbers following consumption of a probiotic *L. rhamnosus* strain DR20 [62]. Lactobacilli and enterococci were detected more frequently (among 10 subjects) and in higher numbers during consumption. Interestingly, the presence of stable populations of lactobacilli before the administration period precluded long-term colonization by the administered probiotic strain [62]. Most subjects ceased shedding the probiotic strain in faeces soon after its consumption stopped, but the *L. rhamnosus* strain remained detectable in faeces of one subject over 2 months after the test period. These data suggest inter-host variables such as bacterium-host or bacterium-diet interactions.

Probiotics and prebiotics are commonly applied in companion animals and production animals [63, 64], and there have been some studies of effects upon the microbiota. Administration of a cocktail containing lactobacilli, bifidobacteria, enterococci, and pediococci improved weight gain in broiler chickens, which was associated with an increase in numbers of *Bifidobacterium* spp., *Lactobacilli*, and Gram-positive cocci [65]. Administration of a probiotic *Enterococcus faecium* strain reduced *E. faecalis* numbers in the intestines of weanling piglets, but total numbers of *E. faecium* remained unchanged, suggesting that the administered strain had displaced part of a fixed number of niche sites occupied by the same species [66]. Many investigations have been published describing the effects of probiotic bacteria on human pathogens (reviewed in [67]), some of which are normal components of production animal microbiota. Enterobacteriaceae numbers were reduced when a cocktail of two *Lactobacillus* strains was administered to pigs [68], and a five-strain probiotic combination reduced *Salmonella enterica* serovar Typhimurium shedding in pigs [69]. Although data from small animal models for human probiotic strains must be interpreted with caution [70], it was interesting to note from a recent study that administration of *L. casei* and *L. plantarum* affected the diversity of murine intestinal lactobacilli, but not the overall bacterial community structure [71]. There was an increase in the number of lactobacilli related to the *acidophilus* complex in the inoculated mice. These animal models provide an opportunity for determining the effect of probiotic administration on the entire microbiota but must ultimately be repeated in humans if that species is the desired host.

Studies in humans are currently few in number, and are often focused in nature. For example, consumption of a commercial probiotic yoghurt reduced *Clostridium difficile*-related diarrhoea in hospitalized patients, but effects on the broader microbiota were not studied [18]. Alterations in the fecal microbiota have been reported in irritable bowel syndrome (IBS) [72, 73]. However, administration of a multispecies probiotic supplementation which alleviated IBS had negligible effect upon the composition of microbiota as measured by quantitative PCR with group-specific primers [74]. However, this approach may have missed changes in microbial composition within these groups. A follow-up study reported stabilization of the microbiota over time [75], which was related to amelioration of symptomatology that was absent from the placebo control group. Alterations of the human intestinal microbiota have also been reported in inflammatory bowel disease (IBD) [76–78]. Given the clinical impetus to find simple non-medicinal solutions to IBD and IBS, one can anticipate renewed vigor in studies of probiotic bacteria as agents for microbiota modulation in these subjects. Probiotics also appear to be efficacious as adjunct therapy for infectious diarrhea, with a recent meta-analysis revealing reduction in risk and duration of diarrhea [79]. Most of the 23 studies included in this analysis were descriptive rather than investigative of the microbiological aspects, and future determination of the effects on the microbiota wrought by probiotic intervention will be very informative. As recently as 2006, the effect of probiotic administration in humans was still being followed by bacteriological culture, but as concluded by the authors of one such study, there was a clear case for culture-independent molecular methods to be applied instead [80]. Community profiling by DGGE showed that lactulose increased the levels of *Bifidobacterium adolescentis* in subjects consuming the prebiotic lactulose, whereas the probiotic yeast *S. boulardii* did not cause any significant universal changes in DGGE profiles [81].

## 5. MECHANISMS OF PROBIOTIC IMPACT ON THE MICROBIOTA

**Figure 1:**
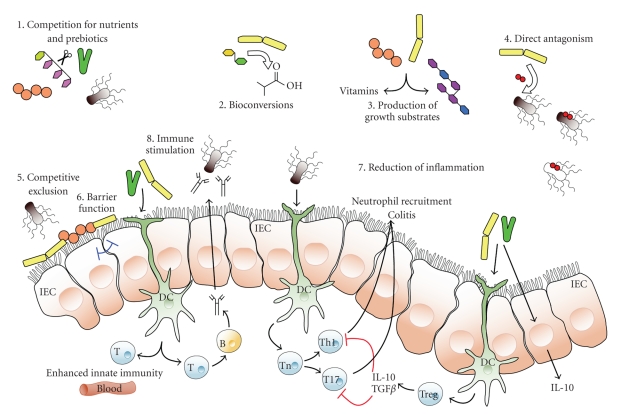
Schematic diagram illustrating potential or known mechanisms whereby probiotic bacteria might impact on the microbiota. These mechanisms include (1) competition for dietary ingredients as growth substrates, (2) bioconversion of, for example, sugars into fermentation products with inhibitory properties, (3) production of growth substrates, for example, EPS or vitamins, for other bacteria, (4) direct antagonism by bacteriocins, (5) competitive exclusion for binding sites, (6) improved barrier function, (7) reduction of inflammation, thus altering intestinal properties for colonization and persistence within, and (8) stimulation of innate immune response (by unknown mechanisms). IEC: epithelial cells, DC: dendritic cells, T:T-cells. For further details, see main text.

Figure 1 shows a schematic overview of the potential mechanisms whereby probiotic micro-organisms might influence the intestinal microbiota. Consumption of probiotic cultures may modulate the microbiota or change its metabolic properties by competition for nutritional substrates. Gordon and colleagues have used transcriptional microarrays to show that introducing a probiotic into the mouse gut changes the way the endogenous microbiota metabolize the diet [82]. When germ-free mice that had been monoassociated with *Bacteroides thetaiotaomicron* were challenged with *Bifidobacterium animalis* or *Lactobacillus casei*, both interventions caused shifts in the gene expression pattern of the *B. thetaiotaomicron* genome [82]. These differentially expressed gene sets (i.e., in response to the two probiotics) did not overlap, emphasizing that different probiotics elicit different responses. However, many of the genes in *B. thetaiotaomicron* whose expression was altered by presence of either probiotic strain were related to expansion of the carbohydrate metabolizing capability of *B. thetaiotaomicron*. Thus, one of the ways in which probiotics can impact upon the composition of the microbiota is apparently by competing with them for substrate availability, and by altering the dynamics of carbohydrate utilization by individual microbiota components. This competition is probably not restricted to the intestine, since recent evidence indicates that oral Bifidobacterium strains (*B. adolescentis*) reduce vitamin K concentration, and may thus compete with *Porphyromonas gingivalis* in the oral cavity [83].

The application of metabolic profiling methods to animal models has suggested another indirect way in which probiotic bacteria might impact on the microbiota, namely, by production of a significantly different microenvironment due to a diverse range of metabolic pathway outcomes. In a recent study using germ-free mice colonized by human baby microbiota and exposed to two lactobacillus strains, Nicholson and colleagues observed microbiome modification, measured by selected culture regimes [84]. This was accompanied by changes in cecal concentrations of short-chain fatty acids, and marked changes in fecal levels of diverse metabolites including choline, acetate, ethanol, a range of putative N-acetylated metabolites (NAMs), unconjugated bile acids (BAs), and tauro-conjugated bile acids. While a natural focus of these studies is the effect of these metabolites upon the host [85], it is likely that such gross changes in metabolic profile also impact upon intestinal microbiota composition. As noted in Table 1, some probiotic bacteria also produce vitamins [27], enhanced availability of which may modulate the microbiota. In addition, exopolysaccharide produced by probiotics including lactic acid bacteria [86] could act as a growth substrate for selected components of the microbiota (see Figure 1).

Probiotic bacteria probably also impact on the general microbiota by direct antagonism. It has been shown in several recent studies that they can modulate numbers of single model organisms in experimental systems. For example, probiotic *L. salivarius* strains inhibit the growth of *H. pylori* *in vitro* in a strain-dependent manner [17], by mechanisms involving lactic acid secretion, and another as yet uncharacterized mechanism (K. A. Ryan and P. W. O'Toole, unpublished). Intestinal *L. salivarius* strains are distinguished by production of a broad-spectrum bacteriocin Abp118 [87], but this is not likely to contribute to antagonism to Gram-negative bacteria like *H. pylori*. However, production of this bacteriocin Abp118 was identified as the mechanism whereby *L. salivarius* UCC118 eliminated *Listeria monocytogenes* infection in a murine model, providing the first definitive mechanism for anti-infective activity of a probiotic bacterium in vivo [16]. Interestingly, both the wild-type strain UCC118 and a bacteriocin-negative derivative were equally able to suppress *Salmonella* Typhimurium infection in the mouse model, suggesting that broader antimicrobial effects on the Gram-negative components of the microbiota may occur. From an opposite perspective, production of a bacteriocin-like substance by vaginal enterococci has been linked to reduction in levels of commensal lactobacilli that is linked to vaginosis [88]. Natural competition between commensals and opportunistic pathogens may therefore be mediated by mechanisms such as bacteriocin production, that can be exploited for using probiotics to modulate the microbiota. Competitive exclusion (see Figure 1), whereby adherent probiotic species occlude access of members of the microbiota to the epithelium [89, 90], represents another way of modulating the microbiota, although strong evidence for this occurring *in vivo* is lacking.

The most subtle effects wrought by probiotics on the microbiota are potentially those that operate by indirect mechanisms involving the host. Improvement of the intestinal epithelium barrier function [91] might theoretically, for example, impact on efficiency of invasion of pathogens, severity of subclinical tissue damage, and release rates of host-derived micronutrients (see Figure 1), that could translate into impacts on the microbiota. In an analogous manner, pathological changes in intestinal epithelium might also favor growth of certain members of the microbiota, if inflamed or damaged epithelial cells differentially affect the microbiota. It is well established that some probiotics can suppress inflammation by inhibiting proinflammatory cytokine production [92–94], and although the molecular basis for this is not currently understood for probiotics, mechanisms and molecules have recently been identified in commensals and pathogens [14, 95]. Reduction in gut inflammation by probiotics could plausibly alter the gut environment sufficiently to impact on the microbiota. Furthermore, some probiotic bacteria have been reported to stimulate the innate immune system both in animal models and in elderly subjects [96, 97], by an unknown mechanism. Administration of probiotic bacteria could thus bolster innate immune activity against transient pathogens, or non-commensal elements in the microbiota, leading to subtle changes in long-term overall composition. However, more studies are required to substantiate the mechanisms in the probiotic-host interactions, and to investigate if they do in fact impact on the microbiota.

## 6. KNOWLEDGE GAPS AND CONCLUDING REMARKS

There has been a rapid recent accumulation of sequence-based information on the composition of the gut microbiota. However, for pragmatic reasons of sample collection facility, this is largely based on fecal analysis, and the microbiota of the colon and small intestine will be different from feces. Studies of the small intestine are particularly warranted because probiotics are proportionally more numerous there, and may exert significant biological activity at this site.

There is adequate information in the literature to support the hypothesis that administration of probiotic cultures in high doses to human subjects will impact on the intestinal microbiota. A comprehensive intervention study, supporting this hypothesis by deep compositional and functional metagenomics approaches, and supplemented by metabolomics, is not currently available (June 2008). In this hypothetical study, mechanisms whereby changes in the microbiota that were achieved could be inferred to a degree by global transcriptional analysis, but definitive linkages between bacterial gene products and effects upon the microbiota could be impossible to establish because of the regulatory issues surrounding human trials with genetically modified organisms. As noted above, proof of principle may be established in animal models, but ultimately these studies must be validated in human subjects. There remains the intriguing question of the role, if any, of the relatively small numbers of potentially probiotic organisms as part of the microbiota in ostensibly healthy individuals. Do these organisms contribute to maintenance of health—or avoidance of disease? Is the level of candidate probiotic organisms in the microbiota critical, and does its importance vary with age? As noted above, there is reasonable evidence that changes in the microbiota accompany disease states like IBD and IBS, conditions whose prevalence increases with aging. There are attractive hygiene-related hypotheses suggesting that depletion of probiotic commensal microbiota in early life may be responsible for the dramatic rise in diseases involving immune dysregulation [98]. The challenge now is to rigorously tackle the interplay of diet, microbiota, and host factors in tractable experiments that will elucidate the key elements in determining outcomes of this interplay, and allow its manipulation.

## Figures and Tables

**Table 1:** Beneficial properties reported for probiotic bacteria.

Host benefit	Microbial trait implicated	Reference^1^
Immune modulation		
* *Stimulation of immunity	Enhance T-cell numbers and activity levels	[13]
* *Dampening of inflammation	Promote anti-inflammatory cytokine production	[14]
Pathogen burden reduction	Competitive exclusion	[15]
	Direct antagonism	[16, 17]
	Uncharacterised	[18, 19]
Improved gut barrier function	Promote gut barrier integrity	[20]
Reduced cancer risk	Detoxification of carcinogenic metabolites	[21]
Reduced atopic allergy symptoms	Suppression of hypersensitivity	[22]
Reduced cardiovascular disease risk	Cholesterol reduction by deconjugation of bile salts	[23, 24]
	Production of anti-hypertensive peptides	[25]
Alleviation of dietary intolerance	Catabolism of dietary ingredients	[26]
Enhanced nutrient value	Vitamin and co-factor production	[27]
Alleviation of IBS^2^ symptoms	Not defined	[28, 29]

^1^Sample reference for each trait. See main text for review references, ^2^Irritable bowel syndrome.
